# Reverse complementary matches simultaneously promote both back-splicing and exon-skipping

**DOI:** 10.1186/s12864-021-07910-w

**Published:** 2021-08-03

**Authors:** Dong Cao

**Affiliations:** Information Processing Biology Unit, Okinawa Institute of Science and Technology Graduate University, 1919-1 Tancha, Onna, Kunigami 904-0495 Okinawa, Japan

**Keywords:** Circular RNA, Back-splicing, Exon-skipping, reverse complementary matches, FACS

## Abstract

**Background:**

Circular RNAs (circRNAs) play diverse roles in different biological and physiological environments and are always expressed in a tissue-specific manner. Especially, circRNAs are enriched in the brain tissues of almost all investigated species, including humans, mice, Drosophila, etc. Although circRNAs were found in *C. elegans*, the neuron-specific circRNA data is not available yet. Exon-skipping is found to be correlated to circRNA formation, but the mechanisms that link them together are not clear.

**Results:**

Here, through large-scale neuron isolation from the first larval (L1) stage of *C. elegans* followed by RNA sequencing with ribosomal RNA depletion, the neuronal circRNA data in *C. elegans* were obtained. Hundreds of novel circRNAs were annotated with high accuracy. circRNAs were highly expressed in the neurons of *C. elegans* and were positively correlated to the levels of their cognate linear mRNAs. Disruption of reverse complementary match (RCM) sequences in circRNA flanking introns effectively abolished circRNA formation. In the *zip-2* gene, deletion of either upstream or downstream RCMs almost eliminated the production of both the circular and the skipped transcript. Interestingly, the 13-nt RCM in *zip-2* is highly conserved across five nematode ortholog genes, which show conserved exon-skipping patterns. Finally, through *in vivo* one-by-one mutagenesis of all the splicing sites and branch points required for exon-skipping and back-splicing in the *zip-2* gene, I showed that back-splicing still happened without exon-skipping, and *vice versa*.

**Conclusions:**

Through protocol optimization, total RNA obtained from sorted neurons is increased to hundreds of nanograms. circRNAs highly expressed in the neurons of *C. elegans* are more likely to be derived from genes also highly expressed in the neurons. RCMs are abundant in circRNA flanking introns, and RCM-deletion is an efficient way to knockout circRNAs. More importantly, these RCMs are not only required for back-splicing but also promote the skipping of exon(s) to be circularized. Finally, RCMs in circRNA flanking introns can directly promote both exon-skipping and back-splicing, providing a new explanation for the correlation between them.

**Supplementary Information:**

The online version contains supplementary material available at 10.1186/s12864-021-07910-w.

## Background

Circular RNAs (circRNAs) are regulatory RNA molecules that are covalently closed by back-splicing, in which a downstream splice donor is joined with an upstream splice acceptor [1]. circRNAs have been reported to bind to microRNAs (the so-called “miRNA sponges”) [2, 3] and to act as decoys for proteins to regulate the expression and function of genes [4–6]. Some circRNAs are translated to functional proteins/peptides through cap-independent mechanisms [7–12]. circRNAs are always expressed tissue-specifically. Especially, circRNAs are enriched in the brain of several species [13–16], like humans, mice, *Drosophila*, etc. In *C. elegans*, circRNAs are also identified [2, 17] and accumulate during aging [18], but such neuronal circRNA profile has not yet been reported.

Back-splicing is a well-regulated process [1]. The reverse complementary matches (RCMs) that locate in the flanking introns of circRNA-producing exons promote circRNA formation by bringing the splice sites for back-splicing to proximity. This model was first proposed when a circular transcript was identified in *Sry* in mice, in which a pair of > 15,500 nt RCMs are present in the introns flanking the ~ 1,200 nt exon to be circularized [19, 20]. Subsequent *in vitro* experiments showed that as less as 400 nt of complementary sequences are sufficient enough for the production of *circSry* [21]. Genome-wide analysis of RNA sequencing (RNA-seq) data in humans revealed that Alu repeats, which contain RCMs, are enriched in the flanking introns of circRNAs [22]. Subsequently, several studies have shown that such RCM sequences in flanking introns promote circRNA formation by using circRNA-expressing vectors [23–26]. More importantly, disturbance of RCMs is shown to be an efficient method for circRNA knockout with little effect on cognate linear mRNA [6, 27]. In *C. elegans*, RCMs are abundant in circRNA-flanking introns [17, 18], but their roles in circRNA production have not been experimentally tested.

circRNA is found to be correlated to exon-skipping [22, 28–32]. In early years, sporadic examples showed that some circRNA-producing genes generate linear transcripts that skip the exons to be circularized [30–32]. Later, systematic analysis of RNA-seq data in human cells found a global correlation of exon-skipping with exon circularization [28]. In *Schizosaccharomyces pombe*, Barrett et al. showed that circRNA could be produced from an exon-containing lariat intermediate produced by exon-skipping [29]. Given that the correlated exon-skipping and circRNA formation use the same pair of introns, it is possible that RCMs in circRNA-flanking introns also regulate exon-skipping.

Here, using *C. elegans* as the model organism, I obtained the neuronal circRNA profile in the L1 stage. Using *zip-2*, a circRNA gene with the correlated skipped transcript, I further investigated RCMs’ roles in the correlation between exon-skipping and back-splicing.

## Results

### Successful neuron isolation for circRNA detection by RNA-seq

**Fig. 1:**
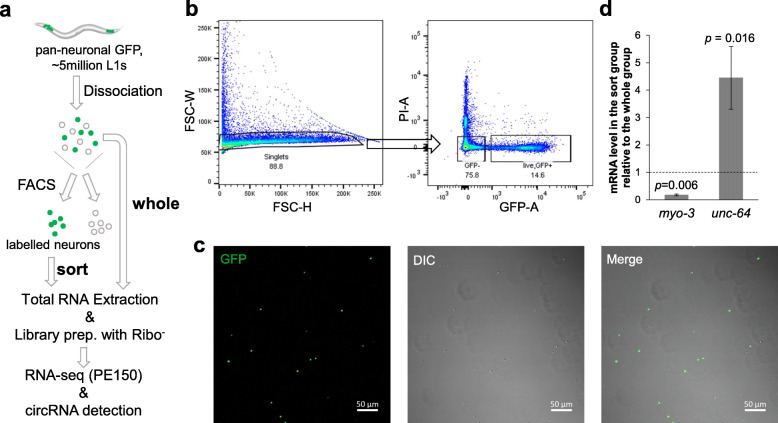
Large scale neuron isolation from *C. elegans* for circRNA detection. **a.** Workflow of neuron isolation and circRNA detection by RNA-sEq. **b.** Gating strategy for FACS: Forward scatter width (FSC-W) was plotted against forward scatter height (FSC-H) to select singlet cells (88.8 %), which were then used for the selection of GFP-positive and PI-negative cells (14.6 %) for sorting. **c.** Confocal images of sorted GFP-positive neurons. Scale bar: 50 μm. **d.** ddPCR results showing the relative levels of two genes (*myo-3* and *unc-64*) in the sort group compared with those in the whole group. Error bars stand for standard deviations of three biological replicates. *P* values are ratio paired *t*-test

Currently, there are no available neuronal circRNA data in *C. elegans*, mainly due to challenges in obtaining enough neuron samples from the tiny worms which have no obviously compartmentalized “brain” tissue. The most common method to obtain neuron cells from *C. elegans* is by the “labeling-dissociation-sorting” method (Fig. 1a) [33–39], in which target neurons are labeled by fluorescent protein and, after mild dissociation of the worms, labeled neurons are collected by fluorescence-activated cell sorting (FACS). This method can obtain target neurons in high purity and is used to detect gene expression in single neurons to all the neurons. However, due to the low efficiency of the dissociation [34, 35], total RNA obtained from sorted cells is limited. Hence this method is only used for mRNA detection, either by microarray or RNA-seq [33, 34, 39–41]. By optimization of previous protocols [35], I aim to improve the final total RNA yield to hundreds of nanograms for circRNA detection by RNA-seq with ribosomal RNA depletion.

Here, using a strain (NW1229) with pan-neuronal green fluorescent protein (GFP) expression, I found that by shortening the time of SDS-DTT treatment (from 2 to 1.5 min) and washing (from 5 × 1.0 min to 5 × 40 s) as well as increasing the time of mechanical disruption (from 10 to 15 min) (Figure S1A), cell yield could be improved. After dissociation, the cell suspension was stained with propidium iodide (PI) to label dead/damaged cells and then subjected to FACS. GFP positive singlet cells were sorted (Fig. 1b). The majority of sorted cells showed GFP fluorescence when observed under a confocal microscope (Fig. 1c). Some neuron cells kept short neurites after sorting (Figure S1B). Consistent with previous findings, neurites can grow out after culture of sorted neuron cells (Figure S1C) [34, 35]. To further confirm the effectiveness of sorting, the levels of two marker genes (*myo-3* and *unc-64*) were quantified by digital droplet PCR (ddPCR). As expected, the neural syntaxin *unc-64* was highly enriched in the sorted cells, whereas the muscle gene *myo-3* was depleted (Fig. 1d).

Using this optimized protocol (see “Methods”), 200–500 ng total RNA was obtained from cells sorted from ~ 1.5–5 million L1 worms (the sort group). RNA samples from dissociated worms before sorting were also prepared for comparison (the whole group, Fig. 1a). For RNA-seq, 150 ng total RNA from three independent trials of the sort group and the whole group was used as input for library preparation with ribosomal RNA removal and first-strand cDNA synthesis using random hexamers. More than 45 million 150 nt paired-end reads were obtained for each sample. Differentially expressed genes between the two groups were analyzed by DESeq2 [42]. Consistent with the ddPCR results (Fig. 1d), *myo-3* was significantly depleted, while *unc-64* was significantly enriched in the sort group compared with the whole group (Figure S1D). The significantly upregulated genes (Table S4) in the sort group were searched in WormExp [43] (https://wormexp.zoologie.uni-kiel.de/wormexp/) to identify whether these genes overlap with previous results of neuronal genes. As expected, the resulted top three datasets were all pan-neural enriched genes determined by microarray analysis of sorted neurons (Figure S1E) [40, 41], indicating the RNA-seq results from sorted samples successfully revealed the gene expression pattern in the neurons.

### circRNAs are highly expressed in the neurons

**Fig. 2:**
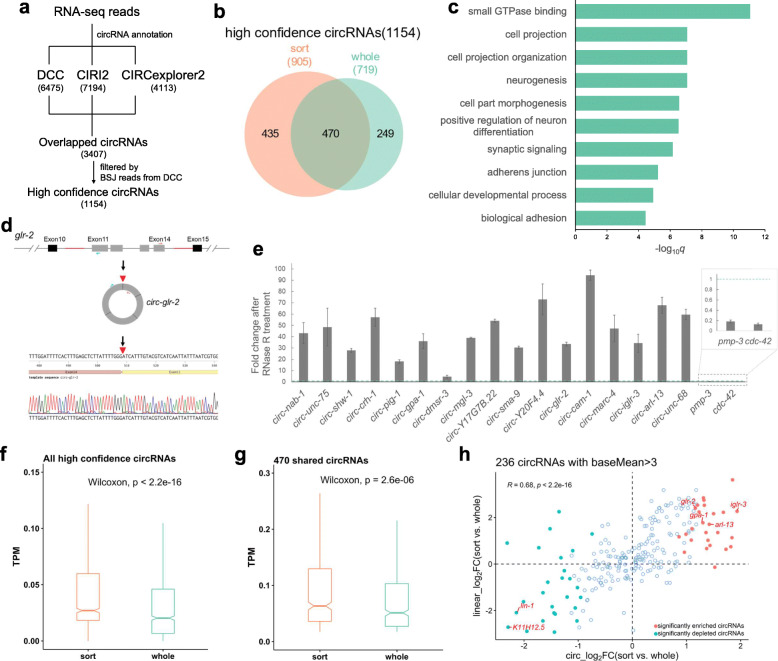
circRNAs are enriched in the neurons of *C. elegans*. **a** Steps to define high-confidence circRNAs used for downstream analysis. Numbers in the brackets are circRNA numbers. **b** Overlap of circRNAs detected in the “sort group” and the “whole group”. **c** Top 10 enriched gene ontology (GO) terms of parental genes of the high-confidence circRNAs. **d** Scheme showing amplification of back-splicing junction of a circRNA from *glr-2* using divergent primers. Amplified sequences are confirmed by Sanger sequencing. The red triangle denotes the joint site. **e** RT-qPCR results of the fold changes of circRNAs and two linear mRNAs (*pmp-3* and *cdc-42*, inset) after RNase R treatment. The blue dashed line shows one-fold change. Error bars are the standard deviations of three biological replicates. **f, g.** TPM (transcripts per million reads) comparison of all circRNAs (**f**) and shared circRNAs (**g**) between the “sort group” and the “whole group”; *p* values are paired Wilcoxon test. **h.** Scatter plot showing the fold changes of 268 circRNAs with baseMean > 3 versus fold changes of their corresponding linear mRNAs. The Pearson correlation coefficient (*R*) and *p* value (*p*) are shown. Significantly differentially expressed circRNAs are shown by colored dots. Names of several circRNA genes are labeled

Combinational use of different circRNA annotation algorithms has shown to reduce false-positive circRNAs [44]. Hence, three methods, DCC [45], CIRI2 [46], and CIRCexplorer2 [47], were used for circRNA annotation from the RNA-seq results, which resulted in 3407 overlapped circRNAs (Fig. 2a and Figure S2A). These circRNAs were further filtered by the back-spliced junction (BSJ) reads from DCC output, with at least three BSJ reads in either the samples of the sort group or the samples of the whole group. This further filtering defined a high-confidence circRNA dataset containing 1154 circRNAs derived from 829 annotated genes and 2 not-annotated loci, which were used for downstream analysis (Fig. 2a and Table S5). Of the 1154 circRNAs, the most majority (96.2 %) of the BSJ reads were from exon-to-exon joining (Figure S2B). The high-confidence circRNAs were compared with a published dataset of circRNAs in aging worms [18], which showed 434 overlapped circRNA (Figure S2C). The novel circRNAs identified here were mainly from the sorted group (Figure S2C), suggesting that sequencing from sorted neuron samples was helpful to identify circRNAs that may not be detected using whole-worm samples. Gene ontology (GO) enrichment analysis of the parental genes of the high-confidence circRNAs showed that terms related to the neuronal functions were significantly enriched, including small GTPase binding, cell projection, neurogenesis, etc. (Fig. 2c).

Two strategies were used to validate the annotated circRNAs: (1) Amplification of the BSJ sequences by RT-PCR using divergent primers followed by Sanger-sequencing (Fig. 2d). Seventeen out of 17 selected circRNAs, including six novel circRNAs, were confirmed with the BSJ sequences (Figure S3). (2) Enrichment quantification by RT-qPCR after RNase R treatment. Since there are no ends in circRNAs, they often show resistance to degradation after treatment with RNase R. As expected, while two linear mRNAs (*pmp-3* and *cdc-42*) were depleted after RNase R treatment, all the circRNAs were enriched (Fig. 2e). The resistance to RNase R was also confirmed by northern blot, which showed that while linear transcript was not detected after RNase R treatment, circRNA from *Y20F4.4* was still detectable (Figure S2D). Together, these results provided supportive evidence of the accuracy of circRNA annotation.

Of the 1154 high-confidence circRNAs, more circRNAs (905/1154) were found in the sort group, with 470 identified in both groups and 249 only in the sort group (Fig. 2b). Next, the abundances of circRNAs in the sort group and the whole group were compared to check whether circRNAs were highly expressed in the neurons of *C. elegans* or not. TPM (transcripts per million reads) values were used for comparison. The principal component analysis (PCA) plot of circRNA TPM showed a clear separation between the two groups (Figure S2E), suggesting different circRNA profiles between them. For all the circRNAs in both groups, circRNAs in the sort group showed significantly higher TPM values than in the whole group (Fig. 2f, *p* < 2.2e-16, paired Wilcoxon test), indicating circRNAs were enriched in the sort group. The same trend was also observed for the shared 470 circRNAs in both groups (Fig. 2g, *p* = 2.6e-6, paired Wilcoxon test).

Next, differentially expressed circRNAs between the sort and the whole group were analyzed, trying to identify neuron-enriched circRNAs. Using BSJ read numbers as input for DESeq2 and filtering with adjusted *p* value < 0.05, 25 circRNAs were found significantly enriched, and 25 circRNAs were significantly depleted in the sort group (Figure S2F, Table S6). Then, I asked whether circRNA levels correlate with the cognate linear mRNA levels. The fold changes of circRNAs between the sort and the whole group were plotted against the fold changes of their cognate linear mRNAs. Here, a cutoff of baseMean (given by DESeq2) bigger than 3 was used, which contained 236 circRNAs, including all the significantly differentially expressed circRNAs (Fig. 2 h). The results showed a strong positive correlation (Fig. 2 h, Pearson’s correlation coefficient *R* = 0.68, *p* < 2.2e-16), which suggested that at the L1 stage of *C. elegans*, neuronal circRNAs were more likely to be derived from highly expressed neuronal genes. When all circRNAs were considered, they still showed a moderately strong positive correlation (Figure S2G, Pearson’s correlation coefficient *R* = 0.5, *p* < 2.2e-16).

### RCMs are required for circRNA production

**Fig. 3:**
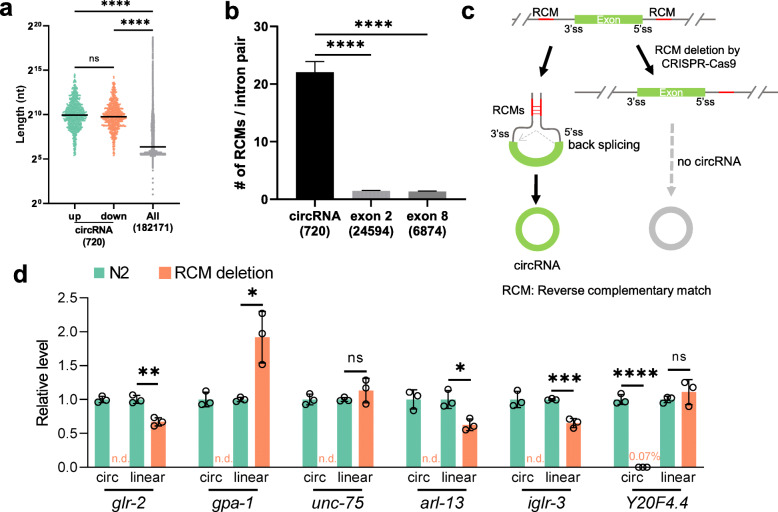
RCMs are required for circRNA production. **a** Length distributions of introns flanking circRNA-producing exon(s), compared with the lengths of all introns. The median values were shown. Numbers in the brackets are numbers of introns used for analysis. **b** Number of RCMs in one pair of flanking introns of circRNA compared with those in control exons (exon 2 and exon 8). Values are shown as mean ± SEM. Numbers in the brackets are numbers of intron pairs used for analysis. *p* values in **a** & **b** are from Kruskal-Wallis test with Dunn’s post-hoc test for multiple comparisons. ****, *p* < 0.0001. **c** Schematic plot showing that RCMs promote circRNA production and the strategy to disturb one of the RCMs by CRISPR-Cas9. **d** Quantification of linear mRNA and circRNA in wild-type N2 strain and RCM deletion mutant strains of six circRNA genes. Error bars are the standard deviations of three biological replicates. n.d.: not detected (Ct values not determined or bigger than those in no-template controls). Two-tail student’s *t*-test. * *p* < 0.05, ** *p* < 0.01, *** *p* < 0.001, **** *p* < 0.0001. ns: not significant

Next, features of circRNA-flanking introns were analyzed. Basic local alignment search tool (BLAST) was used to identify RCMs between each pair of flanking introns using the autoBLAST scripts [18]. Similar to previous findings [17, 18], introns that flank circRNA-producing exons were much longer than average, and much more RCMs were identified when compared with flanking introns of control exons (all exon 2 and exon 8 from annotated genes) (Fig. 3a and b). Best-matched RCMs were also compared, which is the top one hit with the highest “bit score” in the BLAST results of each pair of introns. The average length of the best-matched RCMs in circRNA introns was also much longer than those in introns flanking control exons (Figure S4A).

Although a previous study showed that RCMs could predict the existence of circRNAs [17], the role of RCMs in circRNA formation has not been experimentally confirmed in *C. elegans*. Here, six circRNA genes with RCMs in flanking introns were chosen, and one RCM in each gene was deleted using CRISPR-Cas9 (See “Methods”, Fig. 3c and Figure S4B). Two guide RNAs (gRNAs) that bracket the target RCM were used for each deletion. A 60-nt single-stranded oligo DNA (ssODN) was used as the repair template, with 30 nt homologous sequences in each end (Figure S4C). Which RCM is selected for deletion depends on its position in that intron and the existence of highly specific gRNA sites around RCM sequences. For example, in *glr-2*, the downstream RCM extends to the 3’ splice site, so the RCM in the upstream was chosen for deletion (Figure S4C). The coordinates and lengths of deleted sequences are summarized in Table 1. The gRNA sequences and recombinant single-strand oligo DNAs used for RCM deletions are listed in Table S3. As expected, all the circRNAs were either undetectable or reduced to an extremely low level after the removal of one RCM in the flanking introns (Fig. 3d and Figure S4D), proving that RCMs in *C. elegans* vigorously promote, if not required for, circRNA formation. Of note, in some of the chosen genes, the linear mRNA levels were altered in RCM deletion mutants (Fig. 3d).

**Table 1:** Positions and lengths of deleted RCM sequences in circRNA genes

Gene name	Deleted coordinates	Upstream/Downstream	Deleted length (bp)
*glr-2*	chrIII: 7,142,139–7,142,523	Upstream	385
*gpa-1*	chrV: 11,176,808–11,177,313	Upstream	506
*unc-75*	chrI: 11,592,753–11,593,798	Upstream	1046
*arl-13*	chrI: 2,066,176–2,066,626	Upstream	451
*iglr-3*	chrI: 2,088,411–2,089,756	Downstream	1276
*Y20F4.4*	chrI: 2,034,765–2,035,642	Downstream	878

### RCMs promote both back-splicing and exon-skipping

**Fig. 4:**
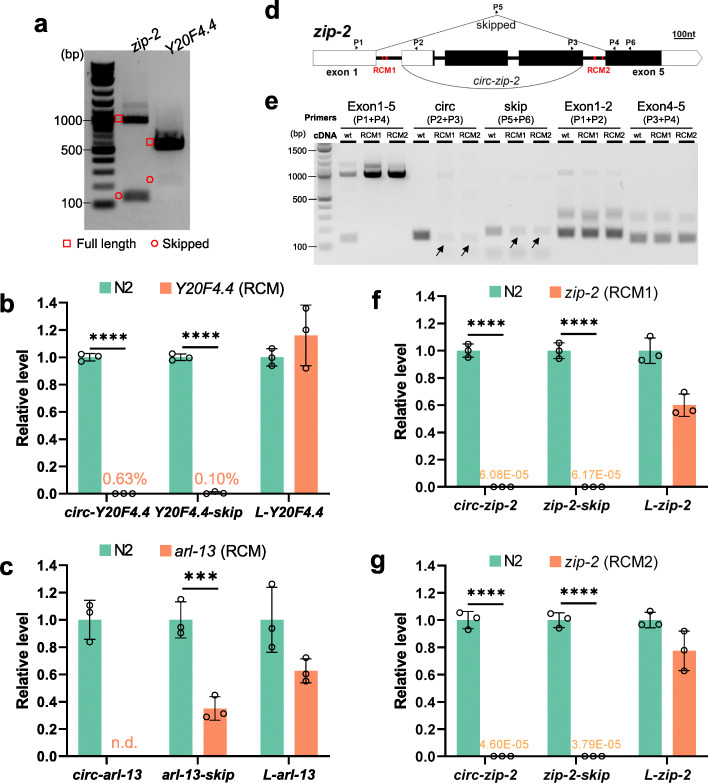
RCMs promote both back-splicing and exon-skipping. **a** Cropped gel image showing RT-PCR detection of full-length transcripts and skipped transcripts in *zip-2* and *Y20F4.4*. **b** RT-qPCR quantification of *Y20F4.4* transcripts in wild-type and RCM-deleted *Y20F4.4* strain. **c** RT-qPCR quantification of *arl-13* transcripts in wild-type and RCM-deleted *arl-13* strain. **d** Illustration of the gene structure of *zip-2*. P1-P6: positions of primers. Black rectangles indicate coding regions and white parts are untranslated regions (UTRs). RCM areas are in red. **e.** Cropped gel image of RT-PCR detection of transcripts from *zip-2* gene in wild-type N2 strain and RCM-deleted strains. **f, g.** RT-qPCR quantification of *zip-2* transcripts in RCM-deleted strains compared with wild-type N2 strain. **b, c, f**, and **g.** Results are normalized to levels in N2 strain using *pmp-3* as the reference gene. Error bars are the standard deviations of three biological replicates. ***, *p* < 0.001, ****, *p* < 0.0001, two-tail Student’s *t-*test

circRNA production has been correlated with exon-skipping that skips the circularized exon(s) [28, 30–32]. In circRNA annotation, DCC also outputs the reads aligned to the correlated skipping junctions for each annotated circRNAs. Of the 1154 high-confidence circRNAs, 330 (29 %) have at least one read aligned to the corresponding skipping junctions, for example, *zip-2* and *Y20F4.4* (Figure S5A and S5B), suggesting the existence of skipped transcripts. For *zip-2* and *Y20F4.4*, RT-PCR using primers that bracket circRNA-producing exon(s) gave two bands, of which the longer ones were full-length transcripts and the shorter ones were confirmed to be the skipped transcripts (Fig. 4a, Figure S5C, and Figure S5E). For some other circRNA genes, the skipped transcripts could be amplified by two-round PCRs, in which the corresponding skipped transcripts were gel-cut purified after first-round PCR, which were used as templates for a second-round PCR (Figure S5D). In total, skipped transcripts were confirmed in 6 out of 7 chosen circRNA genes (Figure S5E).

Previous studies have shown that conserved complementary sequences in introns are associated with exon-skipping [48]. Complementary sequences in different introns regulate mutually exclusive splicing [49–51]. Then whether RCMs are also required for exon skipping is checked. In *Y20F4.4*, the skipped transcript was strongly reduced after removing the upstream RCM (Fig. 4b and Figure S6A). In *arl-13*, the skipped transcript was downregulated in the downstream RCM-deleted mutant (Fig. 4c and Figure S6B). In *zip-2*, two pairs of perfectly matched RCMs, 7 nt and 13 nt in length, respectively, were identified (Fig. 4d and Figure S6C). Deletions of the RCMs in intron 1 or intron 4 were achieved by CRISPR-Cas9 (Figure S6D). Canonical splicings of intron 1 and intron 4 were not affected by RCM deletions (Fig. 4e, Exon 1–2 & Exon 4–5). However, although the circRNA and skipped transcript can be detected in the RCM-deleted strains, their production seemed not as efficient as in the wild-type strain (Fig. 4e, arrows). Quantification of the levels of the three transcripts of *zip-2* (circular, skipped, full-length linear) showed that while full-length linear *zip-2* was only slightly affected, the production of both the circRNA and the skipped transcript was dramatically reduced in both RCM-deleted mutant strains (Fig. 4f g). Together, these findings suggest that RCMs in the flanking introns of circRNA-producing exon(s) also promote the skipping of these exon(s).

### RCM sequences in *zip-2* are highly conserved across several nematode species

**Fig. 5:**
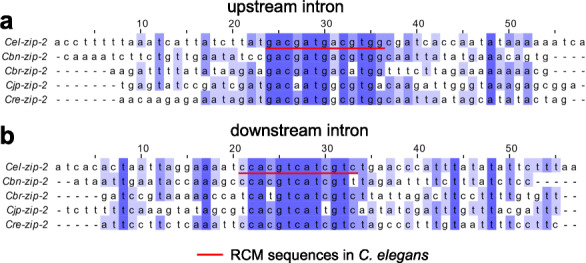
RCM sequences in *zip-2* are highly conserved across several nematode species. **a, b.** Alignment of upstream (**a**) and downstream (**b**) intronic sequences in ortholog *zip-2* genes in indicated nematode species. Red lines underline the 13-nt RCM sequences in *zip-2* of *C. elegans*

Previous studies suggest that competing RNA secondary structures formed by base-pairing between introns that regulate mutually exclusive splicing are evolutionally conserved [52, 53]. I then checked whether RCM sequences in *zip-2* are conserved or not. Ortholog genes of *zip-2* exist in five nematode species (*C. elegans*, *C. brenneri*, *C. briggsae*, *C. japonica*, and *C. remanei*). These *zip-2* genes have similar gene structures (Figure S7). Sequences in the upstream introns and downstream introns of these *zip-2* genes were aligned. Of the two pairs of RCMs in *zip-2* of *C. elegans*, the 13-nt RCMs are highly conserved across the five nematode species, both in the upstream introns and the downstream introns (Fig. 5a and b). Using available splicing data on WormBase, transcripts that skip exons bracketed by the conserved RCMs were found in all these *zip-2* genes (Figure S7, red arrows), suggesting the conserved RCMs possibly promote the conserved exon-skipping in all these *zip-2* genes.

### RCMs do not promote exon-skipping through back-splicing, neither the other way

Current knowledge suggests that RCMs promote circRNA formation by bringing the splicing sites for back-splicing in close proximity. Since the correlated back-splicing and exon-skipping use the same pair of introns, it is possible that RCMs also bring the splice sites for exon-skipping together. In principle, the y-shaped intermediate of back-splicing could be further spliced to form the corresponding skipped transcripts (Fig. 7, back-splicing). Moreover, a previous study has shown that circRNA can be produced through a lariat intermediate produced by exon-skipping [29].

**Fig. 6:**
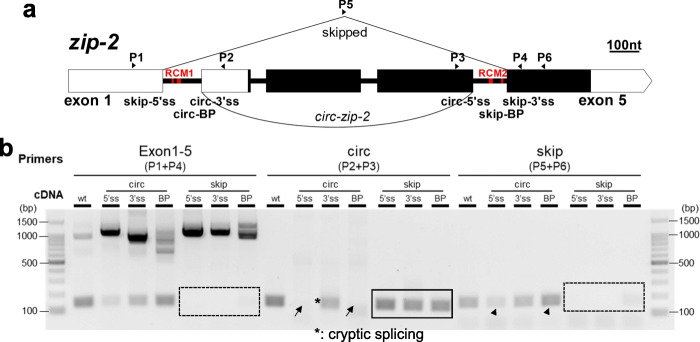
RCMs do not promote exon-skipping through back-splicing, neither the other way. **a** Gene structure of *zip-2*. P1-P6: positions of primers. Positions of splicing sites and branch points that are required for back-splicing and exon-skipping are labeled. Positions of RCMs are in red. **b** Cropped gel image showing RT-PCR detection of *zip-2* transcripts in wild-type N2 strain and strains with mutated ss or BP. Note the cryptic splicing in circ-3’ss strain

**Fig. 7:**
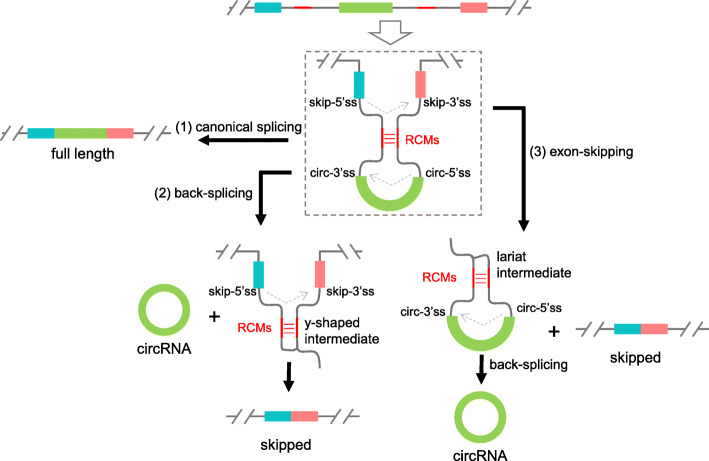
A proposed model that RCMs promote both back-splicing and exon-skipping at the same time. (1) Canonical splicing to form full-length linear mRNA. (2) RCMs facilitate circRNA formation by bringing splice sites for back-splicing sites together. The y-shaped intermediate is further spliced to form the skipped transcript. (3) RCMs promote exon-skipping by bringing splice sites for exon-skipping together. The lariat intermediate is further back-spliced to form circRNA. RCMs in the y-shaped intermediate and the lariat intermediate may help the second splicing steps

Whether RCMs promote exon-skipping first or back-splicing first? There are three possibilities: 1). RCMs promote back-splicing first; 2). RCMs promote exon-skipping first; 3). RCMs promote both back-splicing and exon-skipping at the same time (Fig. 7). In order to clarify the three possibilities, the four splice sites (ss) and two branch points (BP) in intron 1 and intron 4 of *zip-2* were mutated one by one. The 5’ss in intron 1, BP, and 3’ss in intron 4 are used for exon-skipping; hence these sites are named skip-5’ss, skip-BP, and skip-3’ss, respectively. Similarly, BP and 3’ss in intron 1 and 5’ss in intron 4 are named circ-BP, circ-3’ss, and circ-5’ss, respectively (Fig. 6a). For ss mutation, the conserved AG or GT nucleotides were deleted, and some possible cryptic splice sites nearby were mutated (Figure S8A and S8B). For BP mutation, since there is little information about BP sites in *C. elegans* [54], all A nucleotides were changed to G nucleotides in the 3’ half of intron 1 and intron 4, without disturbing the RCM sequences. (Figure S8A and S8B).

The results showed that mutation of ss and BP for exon-skipping sufficiently abolished *zip-2-skip* (Fig. 6b, dashed rectangles). However, *circ-zip-2* was still produced in these mutant strains (Fig. 6b, black rectangle). For mutations of ss/BP required for back-splicing, circ-3’ss mutation produced a circRNA using a noncanonical AA site [55] (Fig. 6b, asterisk, Figure S8B, and S8C). circ-5’ss and circ-BP mutation both blocked circRNA formation (Fig. 6b, black arrows), but the skipped product can still be detected (Fig. 6b, arrowheads). These results suggest that in *zip-2*, exon-skipping is not absolutely required for back-splicing and *vice versa*. RCMs can promote both exon-skipping and back-splicing directly at the same time.

## Discussion

In this study, I optimized a method for large-scale neuron isolation from L1 worms. The amount of obtained RNA from sorted neurons was increased to hundreds of nanogram scale, making the detection of circRNA by RNA-seq more reliable. Using this method, I provided the neuronal circRNA profiles in *C. elegans* and found that circRNAs were abundant in the neurons. Interestingly, circRNAs showing higher levels in the neurons tend to be derived from genes that also show higher expression in the neurons (Fig. 2 h). The time between egg to L1 is the first main period of neuron development, and at the time of hatching, the majority (222/302) of neurons are already formed [56]. The high levels of these circRNAs may be due to the active expression of their parental genes for neuron development at the L1 stage.

RCMs are abundant in circRNA introns of *C. elegans*. I validated that RCMs are required for circRNA formation in multiple circRNA genes. This provides a good method to knockout (KO) circRNAs in *C.* elegans. Especially, RNA interference (RNAi) in *C. elegans* produces secondary short interfering RNAs (siRNAs) that recognize sequences other than the primary targets in the same genes [57], which probably makes circRNA-specific knockdown (KD) by RNAi not working in *C. elegans*. Except for developing the Cas13-based KD method [58] in *C. elegans*, disrupting RCMs sequences may be the only choice to disturb circRNA expression in *C. elegans*. Fortunately, CRISPR-Cas9 based genome editing is quite versatile and of high efficiency in *C. elegans* [59].

The circRNA-KO strains generated in this work did not show any obvious phenotypes in several assays, like locomotion, chemotaxis, lifespan, and aldicarb resistance (data not shown). Even if some phenotypes were observed, they should be carefully interpreted since the linear mRNA levels could also be changed (Fig. 3d).

In *zip-2*, two short pairs of RCMs, 7 nt and 13 nt in length, were identified. To my best knowledge, they are the shortest endogenous *cis* elements that promote circRNA formation. These RCMs were filtered off in the autoBLAST algorithm [18], which was used for global RCM analysis in all circRNA introns. This reminds us that special care is needed to identify *cis* elements that regulate circRNA formation when dealing with specific circRNA genes since such short RCMs are easily neglected. Moreover, the 13-nt RCMs are highly conserved in the *zip-2* ortholog genes in five nematode species, suggesting their roles in promoting exon-skipping and back-splicing may be conserved.

Previous studies of RCMs’ roles in circRNA regulation [25, 26] or splice sites required for back-splicing [24] were mainly based on plasmids in cultured cells. In this work, I show that *C. elegans* is a useful model for *in vivo* investigation of circRNA regulation.

## Conclusions

Currently, two models have been proposed to explain the correlation between exon-skipping and circRNA formation [22, 60]: 1). RCM-promoted back-splicing produces circRNAs and y-shaped intermediates, which are further spliced to form skipped transcripts; 2). Exon-skipping produces skipped transcripts and lariat intermediates, which are further back-spliced to form circRNAs (Fig. 7). The former is used for RCM-driven circRNA genes, and the latter pathway is for circRNA genes that lack RCM sequences. Here, I show that RCMs are not only required for back-spicing but also promote exon-skipping. I further delineated that RCMs are not promoting exon-skipping through back-splicing, neither the other way. Instead, the two pathways are happening together, possibly competing with each other. I propose that RCMs in the introns not only bring the splice sites for back-splicing to proximity but also bring the sites for exon-skipping together, facilitating both processes simultaneously. Since the RCMs still exist in the intermediates of back-splicing and exon-skipping, they may function twice to promote further splicing/back-splicing in these intermediates (Fig. 7).

## Methods

### Worm maintenance

*C. elegans* Bristol N2 strain was used as the wild type. Worms were maintained using standard conditions on Nematode Growth Media (NGM) agar plates with *Escherichia coli* strain OP50 [61] at 20°C or 25^o^C. Strains used in this study are listed in Table S1.

### Worm synchronization

Worm synchronization was performed by bleaching for large-scale worm preparation (L1 worms for dissociation). Briefly, worms were washed off plates using M9 buffer when a lot of eggs were laid and most of the worms were gravid adults. The worms were washed with M9 buffer and then bleached in bleach solution (1 M NaOH, 0.6 % (m/v) NaClO) with ~ 5 min continuous shaking. Then eggs were pelleted and washed three times with 12 ml M9 buffer by centrifuging at 2000 rpm for 0.5 min. Finally, the egg pellet was re-suspended in ~ 5 ml M9 buffer and rocked at room temperature for 17–24 h to hatch.

### L1 worm dissociation

To ~ 80 µl of L1 worm pellet, 200 µl SDS-DTT solution (200 mM DTT, 0.25 % SDS, 20 mM HEPES pH 8.0, 3 % sucrose) was added, followed by a 1.5-minute incubation at room temperature. Then, the worm pellet was washed 5x with 1 ml egg buffer (25 mM HEPES pH 7.3, 118 mM NaCl, 48 mM KCl, 2 mM CaCl_2_, 2 mM MgCl_2_, 0.340 ± 0.005 Osmolarity) and centrifuged at 10,000 × g for 30 s. The washing steps and centrifugation should be performed quickly so that one round of washing and centrifugation is done in 40–50 s. The washed worm pellet was re-suspended in 100 µl pronase (15 mg/ml in egg buffer) from *Streptomyces griseus* (Sigma-Aldrich). Worms were dissociated by periodic mechanical disruption by pipetting for 15 min. 200 µl tips were used for mechanical disruption by the method mentioned in Zhang et al.’s protocol (“Pipette the larvae suspension with a 200 µl tip during the digestion. Adjust the pipetting volume to the approximate volume of the suspended pellet. Slowly pull suspended larvae into the pipette tip. Then, press down to force the pipette tip against the bottom of the microcentrifuge tube and slowly eject the contents”) [35]. Do as many times as possible. When most worm bodies were dissociated, 900 µL L-15/FBS medium (10 % FBS in Leibovitz’s L-15 medium (Gibco), 0.340 ± 0.005 Osmolarity adjusted by sucrose) was added. Cells were collected and washed twice with 1 ml egg buffer by centrifuging at 9600 × g for 5 min at 4^o^C. Cells were suspended in the appropriate amount of egg buffer and allowed to sit on ice for at least 30 min. The upper volume of cell suspension was used for FACS. For whole worm control, after dissociation and washing, the cell suspension was put on ice in the whole procedure of sorting.

### Fluorescence-Activated Cell Sorting (FACS)

Sorting was performed on a FACS AriaII flow cytometer (Becton Dickinson) equipped with a 70 μm nozzle. 2 and 3.4 μm polystyrene beads (Spherotech) were used for size calibration. Before sorting, propidium iodide (PI) was added to the cell suspension to a final concentration of 0.2 – 0.5 µg/ml. Then, profiles of dissociated cells from GFP-labeled strains were compared to profiles of cells from N2 worms to exclude auto-fluorescent cells. Sorted cells were collected in 3 ml L-15/FBS medium in a 15 ml conical tube chilled on ice. For RNA extraction, sorted cells and whole worm control samples were collected by centrifugation in a swing-bucket centrifuge at 4400 rpm, 4^o^C for 10 min. The supernatant was removed and 0.3 ml Trizol solution (Invitrogen) was added and stored at -80^o^C. For culture, sorted cells were seeded onto a poly-D-lysine coated glass-bottom dish (MatTec) with daily changes of L-15/FBS buffer. Cells were visualized by confocal microscopy (Carl Zeiss, LSM780) with a 60 × oil lens.

### Mutagenesis by CRISPR-Cas9

Mutation by CRISPR-Cas9 was based on the protocol published by Dokshin et al. [62] with minor modifications. Briefly, Cas9 (Sigma-Aldrich; 0.5 µl, 10 µg/µl in supplied buffer), tracrRNA (Sigma-Aldrich; 5 µl, 0.4 µg/µl in 10 mM Tris-HCl, pH7.5) and designed crRNA (ThermoFisher or IDT; 0.4 µg/µl in Tris-HCl, pH7.5; 2.8 µl for single crRNA, 1.4 µl each for 2 crRNAs) were mixed and incubated at 37^o^C for 10 min. Then recombinant dsDNA fragment (add to > 400 ng/µl final concentration) or recombinant single-strand DNA (ordered from Invitrogen or IDT; 2.2 µl, 1 µg/µl in Tris-HCl, pH7.5), injection marker pRF4(*rol-6*) (2.7 µl, 300 ng/µl in Tris-HCl, pH7.5), KCl (0.5 µl, 1 M), HEPES (1 µl, 0.2 M, pH7.4) and H_2_O were added to make a final 20 µl injection mixture. Injected P0 worms were recovered at 20^o^C overnight and then transferred to RT (25^o^C). For F1 with obvious phenotypes, F1 worms with target phenotype were picked, and homozygous progeny were kept. For mutagenesis with no obvious genotypes, ~ 10 F1 rollers were picked onto separated plates, and their genotypes were checked by single worm PCR after laying eggs. Large indels were identified by amplicon size differences. Small indels were checked by enzyme digestion of amplicons. Non-roller homozygous progenies with target genotype were kept. CrRNAs, recombinant ssODNs, validation primers, and restriction enzymes used in this work are listed in Table S3.

### RNA extraction

RNA extraction from sorted samples and whole worm samples was performed using Direct-zol RNA MicroPrep kit (ZYMO Research) with on-column DNase I (ZYMO Research) digestion according to the manufacturer’s protocol. RNA quality and quantity were measured by High Sensitivity RNA ScreenTape (Agilent) on TapeStation 4200 (Agilent). For RNA extraction from worms, worms were first flash-frozen in Trizol solution (Invitrogen) in liquid N_2_ and then homogenized in by vortexing with glass beads (φ 0.1 mm) in Beads Cell Disrupter MS-100 (TOMY). If not mentioned, all the RNA samples used in this study were from L1 worms of indicated genotypes.

### RNA Sequencing

For RNA-seq of samples from sorted neurons (the sort group) and whole worms (the whole group), libraries were prepared using KAPA RNA HyperPrep kit with RiboErase (HMR) (KAPA biosystems) according to the manufacturer’s protocol. The RNA input was 150 ng and fragmentation conditions were 85^o^C for 5 min. Barcodes were introduced to each sample using KAPA duel-indexed adapters (KAPA biosystems). Length distribution of each library was determined by TapeStation 4200 (Agilent) using High Sensitivity DNA ScreenTape (Agilent). Libraries were quantified by KAPA library quantification kit (KAPA biosystems) and then multiplexed and sequenced on Illumina Hiseq 4000 platform to obtain 150 nt paired-end reads.

### Droplet digital PCR (ddPCR)

cDNA was reverse transcribed from 10 ng total RNA using an iScript Advanced cDNA synthesis kit (Bio-Rad). ddPCR was performed by using ddPCR EvaGreen Supermix kit (Bio-Rad) on a QX200 Droplet Reader (Bio-Rad) based on the manufacturer’s protocol. Results were analyzed using QuantaSoft software (Bio-Rad).

### Real-time PCR

Real-time PCR reactions were performed using soAdvanced Universal SYBR Green Supermix (Bio-Rad) with cDNAs synthesized from iScript Advanced cDNA synthesis kit (Bio-Rad). 20 µl reaction mix with 2 µl cDNA (~ 1–10 ng) were monitored on StepOnePlus Thermal Cycler (Applied Biosystems) in “fast mode”. Cycling conditions: 95^o^C, 30’, 40 or 45 cycles of 95oC, 15’ and 60^o^C, 30’ with plate reading, and a final melt curve stage using default conditions. Primers used are listed in Table S2.

### RNase R treatment

Total RNA was treated with or without (Mock) RNase R (2 U/µg) in the presence of Ribolock (2 U/µg) (ThermoFisher Scientific). The reaction was incubated at 37^o^C for 30 min. Then RNA was purified with an RNA Clean and Concentrator kit (ZYMO Research) according to the manufacturer’s protocol. For fold change quantification, RNA was quantified by Nanodrop and an equal amount of RNA input was used for cDNA synthesis. Fold changes were calculated by 2^(Δ−Ct)^ between the paired samples. For northern blot, 20 µg total RNA with or without RNase R treatment was used for loading.

### Northern blot

Northern blot was performed using NorthernMax kit (ThermoFisher Scientific) and the probes were labeled by α-^32^P-deoxycytidine 5’-triphosphate (PerkinElmer) using Random Primer DNA Labeling Kit Ver. 2 (Takara, #6045) according to manufacturer’s protocols. Briefly, RNA samples (10 or 20 µg) were resolved in 1 % agarose gel by electrophoresis at 5 V/cm in 1× MOPS buffer for ~ 2 h. Then RNA was transferred onto an Amersham Hybond-N + membrane (GE Healthcare) by capillary blot for 2.5 h using the transfer buffer supplied in NorthernMax kit. Transferred RNA was crosslinked by 254 nm UV at 1200 × 100 µJ/cm^2^ (Analytik Jena CL-1000). Prehybridization was performed in ULTRAHybe buffer at 50^o^C for one hour, followed by hybridization with ^32^P labeled probes overnight at 50^o^C. The membrane was washed 2 × 5 min at room temperature using Low Stringency Washing Solution and 2 × 15 min at 50^o^C using High Stringency Washing Solution. The membrane was sealed in kitchen wrap and exposed to a phosphorscreen for several hours to overnight, and the signals were detected by Typhoon FLA7000 (GE Healthcare). Primers used for probe amplification are listed in Table S2.

### circRNA prediction and RNA-seq data analysis

DCC [45], CIRI2 [46], and CIRCexplorer2 [47] were used for circRNA annotation from RNA-seq data. For DCC, raw reads were aligned to reference genome (WBcel235/ce11) using STAR [63] (https://github.com/alexdobin/STAR) with the following options: --outSJfilterOverhangMin 15 15 15 15 –alignSJoverhangMin 15 –alignSJDBoverhangMin 15 --outFilterScoreMin 1 --outFilterMatchNmin 1 --outFilterMismatchNmax 2 --chimSegmentMin 15 --chimScoreMin 15 --chimScoreSeparation 10 --chimJunctionOverhangMin 15. Then the output files from STAR, chimeric.out.junction, were used for circRNA annotation with DCC (https://github.com/dieterich-lab/DCC). For CIRI2, RNA-seq reads were aligned to WBcel235/ce11 genome by BWA with the following scripts (using sort_1 as an example):

bwa mem -T 19 -t 64 /path/to/genome.fa sort_1_R1_001.fastq.gz sort_1_R2_001.fastq.gz > sort_1.sam

perl ./CIRI2.pl -I ./sort_1.sam -O sort_1_all -F /path/to/genome.fa -A /path/to/genes.gtf -T 12 − 0

For CIRCexplorer2, RNA-seq reads were aligned using STAR with the following option: --chimSegmentMin 10. Then annotation was performed following the recommended conditions in the manual (https://circexplorer2.readthedocs.io/en/latest/). Overlapped circRNAs were obtained by comparing circRNA coordinates identified in each method. Differential expression analyses of mRNAs and circRNAs were performed using DESeq2 [42] package in R with the gene count output from STAR or the BSJ junction count output from DCC, respectively. The plots (PCA plots, boxplots, scatter plots) were generated using ggplot2 package (https://ggplot2.tidyverse.org/) and ggpubr (http://www.sthda.com/english/rpkgs/ggpubr) package in R.

### RCM analysis

RCM analysis in flanking introns of circRNAs or control exons was performed using IntronPicker and autoBLAST scripts (https://github.com/alexandruioanvoda/) described in [18].

### Microscopy

Confocal images were obtained using a Zeiss LSM780 confocal microscope, and images were processed using the ZEISS ZEN3.1 software.

### Gene ontology analysis

Gene ontology enrichment analysis was performed using WormBase Enrichment Suite webserver [64, 65] (https://wormbase.org/tools/enrichment/tea/tea.cgi).

### Statistical analysis

Statistical analysis was performed using R or Prism (GraphPad).

## Supplementary Information

**Additional file 1: Figure S1. **Dissociation and sorting of L1 worms. (A) Steps of L1 worm preparation and dissociation for FACS. Optimized conditions are in bold. (B) Representative confocal image of sorted cells. Note the short neurites (red rectangles) of some cells. Scale bar: 20 μm. (C) Confocal images showing sorted neurons after five-day culture at 20^o^C. Scale bars are 5 μm. (D) Volcano plot showing differentially expressed genes between the sort group and the whole group. *myo-3* and *unc-64* are labeled. (E) Output from WormExp for gene set enrichment search using upregulated genes in the neurons in our dataset. Red rectangle highlights the top 4 hits. **Figure S2. **circRNA analysis and experimental validation. (A) Overlap of circRNAs annotated by three algorithms: DCC, CIRI2, and CIRCexplorer2. (B) Ratios of back-splicing junction types of the high-confidence circRNAs. (C) Overlap of high-confidence circRNAs in this work and filtered circRNAs in work of Cortés-López et al (*1*). (D)Northern blot detection of *Y20F4.4* transcripts in total RNA (20 μg) without or with RNase R treatment, using probes that hybridize to both linear and circular transcripts. Theoretical lengths of the linear and circular transcripts were labeled. The blot image is cropped for clarity and the full image is in Additional file 1, Figure S9. (E) PCA plot of circRNAs in the sort group and the whole group. (F) MA plot showing differentially expressed (DE) circRNAs between the sort group and the whole group. Significantly DE circRNAs are highlighted by colors. The gene names of some circRNA genes are labeled. (G) Scatter plot showing the correlation of log2 fold change of circRNAs and their cognate linear RNAs in the sort group and whole group. The Pearson correlation coefficient (*R*) and *p* value (*p*) are shown. Significantly DE circRNAs are shown by colored dots. **Figure S3.** Sanger sequencing results of the BSJ sequences of selected circRNAs. Red triangles denote the joint sites. **Figure S4. **circRNA-flanking intron analysis and RCM deletions in circRNA genes. (A) Lengths of best-matched RCMs in one pair of circRNA-flanking introns compared with those in control exons (exon 2 and exon 8). Values are shown as mean ± SEM. Numbers in the brackets are numbers of intron pairs for analysis. *p* values are from Kruskal-Wallis test with Dunn’s post-hoc test for multiple comparisons. ****, *p* < 0.0001. (B) Positions of deleted RCMs (red line) of the 6 circRNA genes. Exons in orange shadows are to form circRNAs. (C) Illustration of RCM deletion in *glr-2*. Red lines in introns are RCMs. Red crosses denote gRNA positions. (D) Cropped gel image of amplification of circRNA using divergent primers in wild-type N2 strain and RCM-deletion strains (mutant) of 6 circRNA genes. **Figure S5. **Skipped transcripts in several circRNA genes. (A & B) Sashimi plots showing the number of reads that aligned to the junction of back-splicing, canonical splicing, and exon-skipping in *zip-2* and *Y20F4.4* in the RNA-seq dataset of this study. Exon(s) in red rectangles are circularized to form circRNAs. (C) Illustration of a circRNA-producing gene producing three transcripts: full-length, circular and skipped. Primers used to detect both the full-length and the skipped transcripts are shown. (D) Amplification of the skipped transcripts from several circRNA genes by 2-round PCRs. Red rectangles mark the gel areas to be cut. Gel images were cropped for clarity. (E) Confirmation of sequences of the skipped transcripts in 6 circRNA genes. **Figure S6. **Effect of RCM-deletion on exon-skipping. (A) RT-PCR detection of *Y20F4.4* transcripts in wild-type N2 strain (wt) and the RCM-deleted *Y20F4.4*strain (mut). Gel image was cropped for clarity. (B) RT-PCR detection of *arl-13*transcripts in wild-type N2 strain (wt) and the RCM-deleted *arl-13*strain (mut). Gel image was cropped for clarity. (C) Folding prediction of intron 1 and intron 4 of *zip-2* by Mfold (http://www.unafold.org/mfold/applications/rna-folding-form.php). RCM sequences are highlighted. (D) Deleted RCM sequences in intron 1 and intron 4 of *zip-2*. **Figure S7. **Gene structures of *zip-2* ortholog genes.Gene structures of ortholog *zip-2* genes in indicated nematode species are shown. The splicing patterns of these genes are also shown (from WormBase), with rectangles indicating the joining of 5’ss and 3’ss. The conserved 13-nt RCMs are labeled. Red arrows indicate the splice junctions of the skipped transcripts. Note that the RCMs are always near the joining sites of exon-skipping in these genes. There are two copies of RCM sequences in the upstream intron of *Cbr-zip-2*. **Figure S8. **Sequence confirmation of mutated ss and BP sites in *zip-2*. (A, B) Sanger sequence results of splicing sites and branch points mutation in intron 1 and intron 4 of *zip-2*. The enzyme digestion sites used to distinguish wild-type sequences and mutated sequences are labeled. The position of the cryptic 3’ss in circ-3’ss mutation is labeled. (C) Sanger sequence of *circ-zip-2* produced from the *zip-2*(circ-3’ss) strain. Note that amplified sequences are 2 nt shorter than the predicted BSJ sequences. **Figure S9.** Original full images of northern blot and agarose gels. The corresponding figure numbers are labeled. Kept areas are in red rectangles.

**Additional file 2: Table S1.** Strains used in this study.

**Additional file 3: Table S2.** Primers used in this study.

**Additional file 4: Table S3.** List of gRNA sequences, recombinant oligos, and validation primers used for mutagenesis by CRISPR-Cas9.

**Additional file 5: Table S4.** Differential expression analysis results of linear mRNAs between the sort and the whole group by DESeq2.

**Additional file 6: Table S5.** List of high-confidence circRNAs with numbers of BSJ reads in each sample.

**Additional file 7: Table S6.** Differential expression analysis results of the high-confidence circRNAs between the sort and the whole group by DESeq2.

## Data Availability

Raw FASTQ files from the RNA-seq data were deposited at the NCBI Sequence Read Archive (BioProject: PRJNA669379; https://www.ncbi.nlm.nih.gov/bioproject/PRJNA669379). All strains and other materials are available upon request.

## Abbreviations

*C. elegans*: *Caenorhabditis elegans*
Ct: Threshold cycle
L1: The first larval
RCMs: Reverse complementary matches
circRNA: Circular RNA
FACS: Fluorescence-activated cell sorting
PI: Propidium iodide
ddPCR: Digital droplet PCR
cDNA: Complementary DNA
RT-qPCR: Quantitative reverse transcription PCR
GO: Gene ontology
BSJ: Back-spliced junction
TPM: Transcripts per million reads
PCA: Principal component analysis
FC: Fold change
gRNA: Guide RNA
ss: Splice site
BP: Branching point
SEM: Standard error of the mean
UTR: Untranslated region
KO: Knockout
KD: Knockdown
