# Supplementary material for: Reverse complementary matches simultaneously promote both back-splicing and exon-skipping
Source: BMC Genomics. 2021 Aug 3;22:586. doi: 10.1186/s12864-021-07910-w (PMC8330042; doi:10.1186/s12864-021-07910-w)
Supplement: Supplementary file 1 — Additional file 1: Figure S1. Dissociation and sorting of L1 worms. (A) Steps of L1 worm preparation and dissociation for FACS. Optimized conditions are in bold. (B) Representative confocal image of sorted cells. Note the short neurites (red rectangles) of some cells. Scale bar: 20 μm. (C) Confocal images showing sorted neurons after five-day culture at 20oC. Scale bars are 5 μm. (D) Volcano plot showing differentially expressed genes between the sort group and the whole group. myo-3 and unc-64 are labeled. (E) Output from WormExp for gene set enrichment search using upregulated genes in the neurons in our dataset. Red rectangle highlights the top 4 hits. Figure S2. circRNA analysis and experimental validation. (A) Overlap of circRNAs annotated by three algorithms: DCC, CIRI2, and CIRCexplorer2. (B) Ratios of back-splicing junction types of the high-confidence circRNAs. (C) Overlap of high-confidence circRNAs in this work and filtered circRNAs in work of Cortés-López et al (1). (D)Northern blot detection of Y20F4.4 transcripts in total RNA (20 μg) without or with RNase R treatment, using probes that hybridize to both linear and circular transcripts. Theoretical lengths of the linear and circular transcripts were labeled. The blot image is cropped for clarity and the full image is in Additional file 1, Figure S9. (E) PCA plot of circRNAs in the sort group and the whole group. (F) MA plot showing differentially expressed (DE) circRNAs between the sort group and the whole group. Significantly DE circRNAs are highlighted by colors. The gene names of some circRNA genes are labeled. (G) Scatter plot showing the correlation of log2 fold change of circRNAs and their cognate linear RNAs in the sort group and whole group. The Pearson correlation coefficient (R) and p value (p) are shown. Significantly DE circRNAs are shown by colored dots. Figure S3. Sanger sequencing results of the BSJ sequences of selected circRNAs. Red triangles denote the joint sites. Figure S4. circRNA-f [file 12864_2021_7910_MOESM1_ESM.docx]

**Additional file 1**


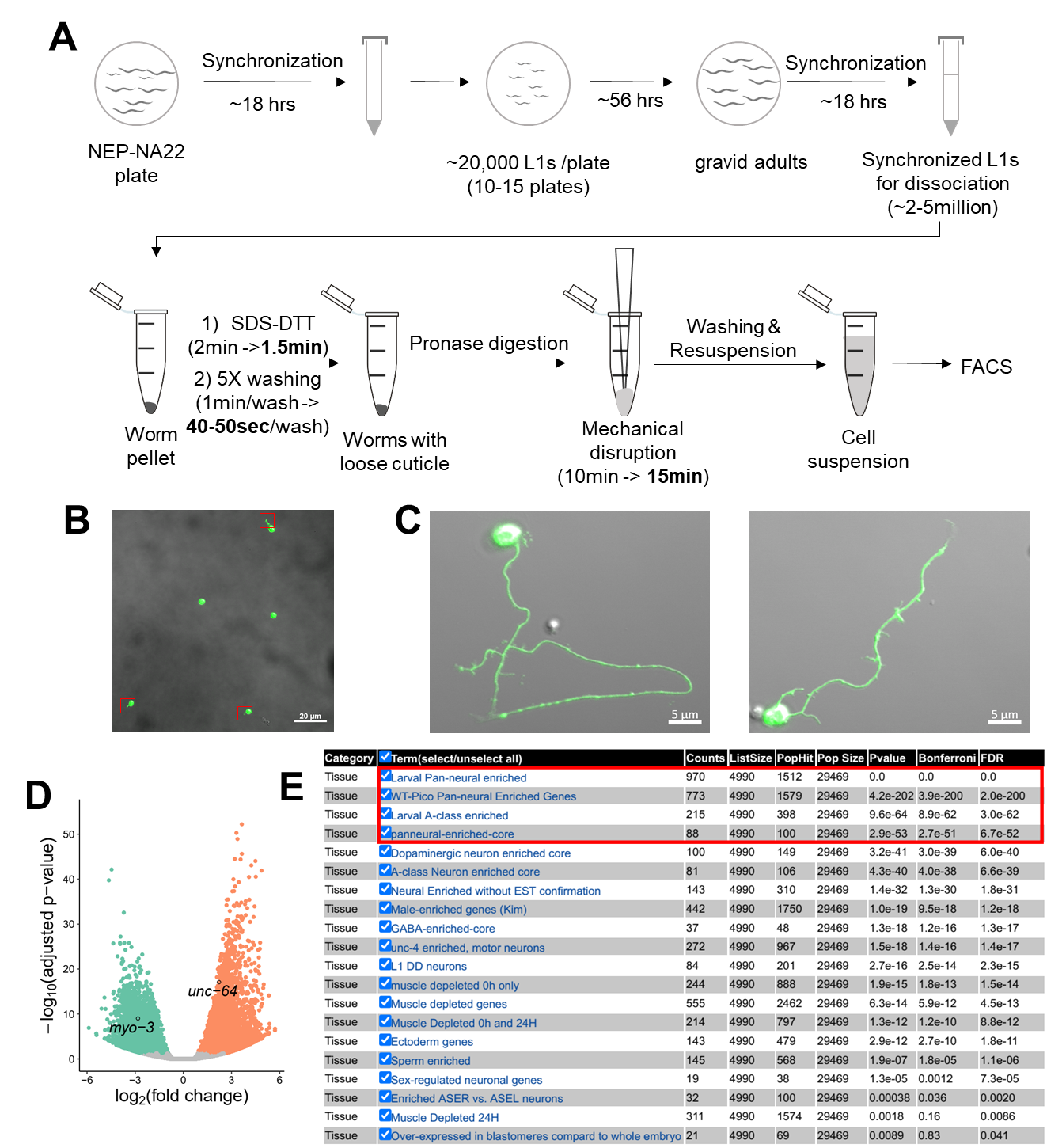


**Figure S1.** Dissociation and sorting of L1 worms.

(A) Steps of L1 worm preparation and dissociation for FACS. Optimized conditions are in bold.

(B) Representative confocal image of sorted cells. Note the short neurites (red rectangles) of some cells. Scale bar: 20 μm.

(C) Confocal images showing sorted neurons after five-day culture at 20^o^C. Scale bars are 5 μm. (D) Volcano plot showing differentially expressed genes between the sort group and the whole group. *myo-3* and *unc-64* are labeled.

(E) Output from WormExp for gene set enrichment search using upregulated genes in the neurons in our dataset. Red rectangle highlights the top 4 hits.


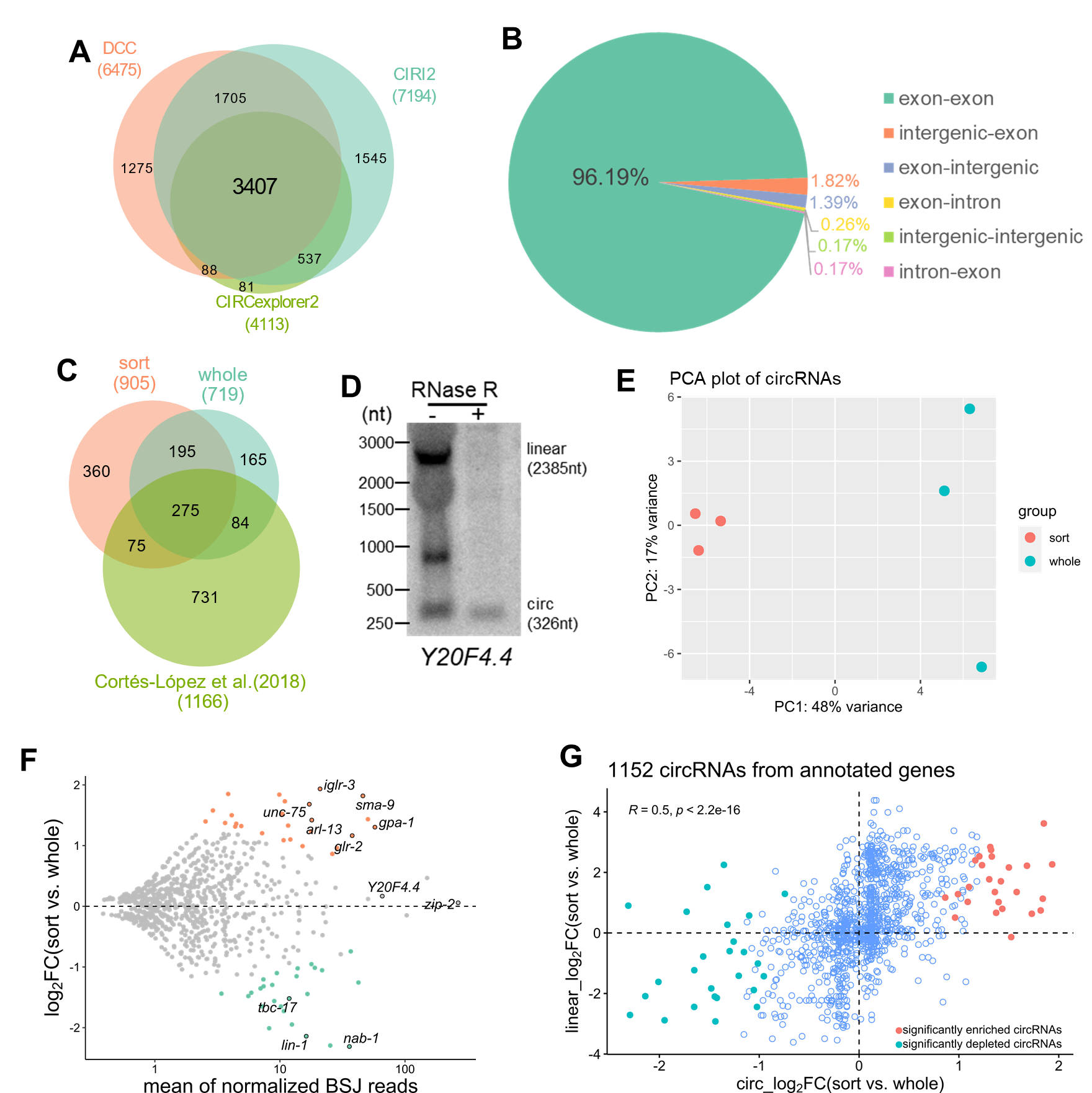


**Figure S2.** circRNA analysis and experimental validation.

(A) Overlap of circRNAs annotated by three algorithms: DCC, CIRI2, and CIRCexplorer2.

(B) Ratios of back-splicing junction types of the high-confidence circRNAs.

(C) Overlap of high-confidence circRNAs in this work and filtered circRNAs in work of Cortés-López et al (*1*).

(D)Northern blot detection of Y20F4.4 transcripts in total RNA (20 μg) without or with RNase R treatment, using probes that hybridize to both linear and circular transcripts. Theoretical lengths of the linear and circular transcripts were labeled. The blot image is cropped for clarity and the full image is in Additional file 1, Figure S9

(E) PCA plot of circRNAs in the sort group and the whole group.

(F) MA plot showing differentially expressed (DE) circRNAs between the sort group and the whole group. Significantly DE circRNAs are highlighted by colors. The gene names of some circRNA genes are labeled.

(G) Scatter plot showing the correlation of log2 fold change of circRNAs and their cognate linear RNAs in the sort group and whole group. The Pearson correlation coefficient (*R*) and *p* value (*p*) are shown. Significantly DE circRNAs are shown by colored dots.


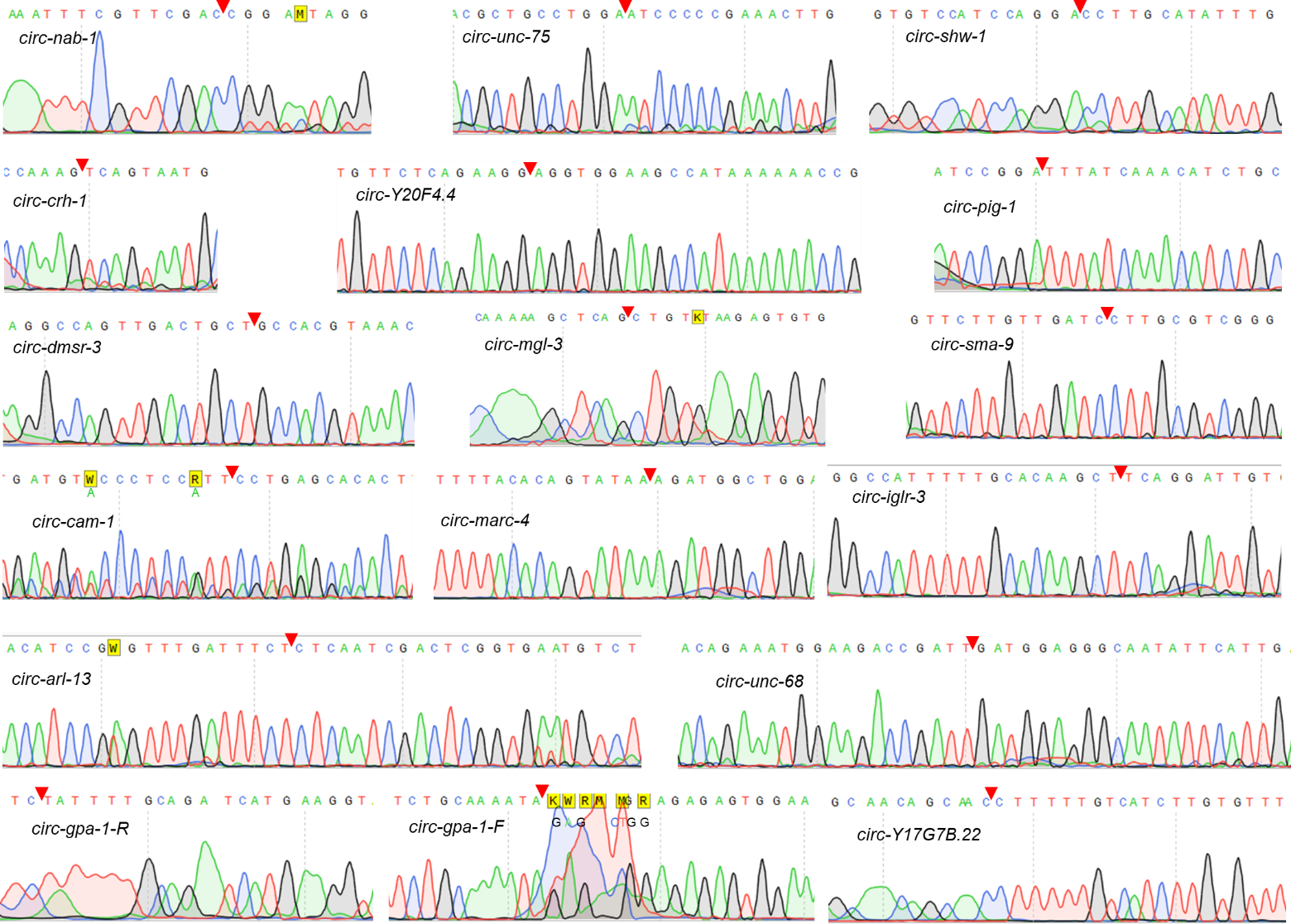


**Figure S3.** Sanger sequencing results of the BSJ sequences of selected circRNAs. Red triangles denote the joint sites.


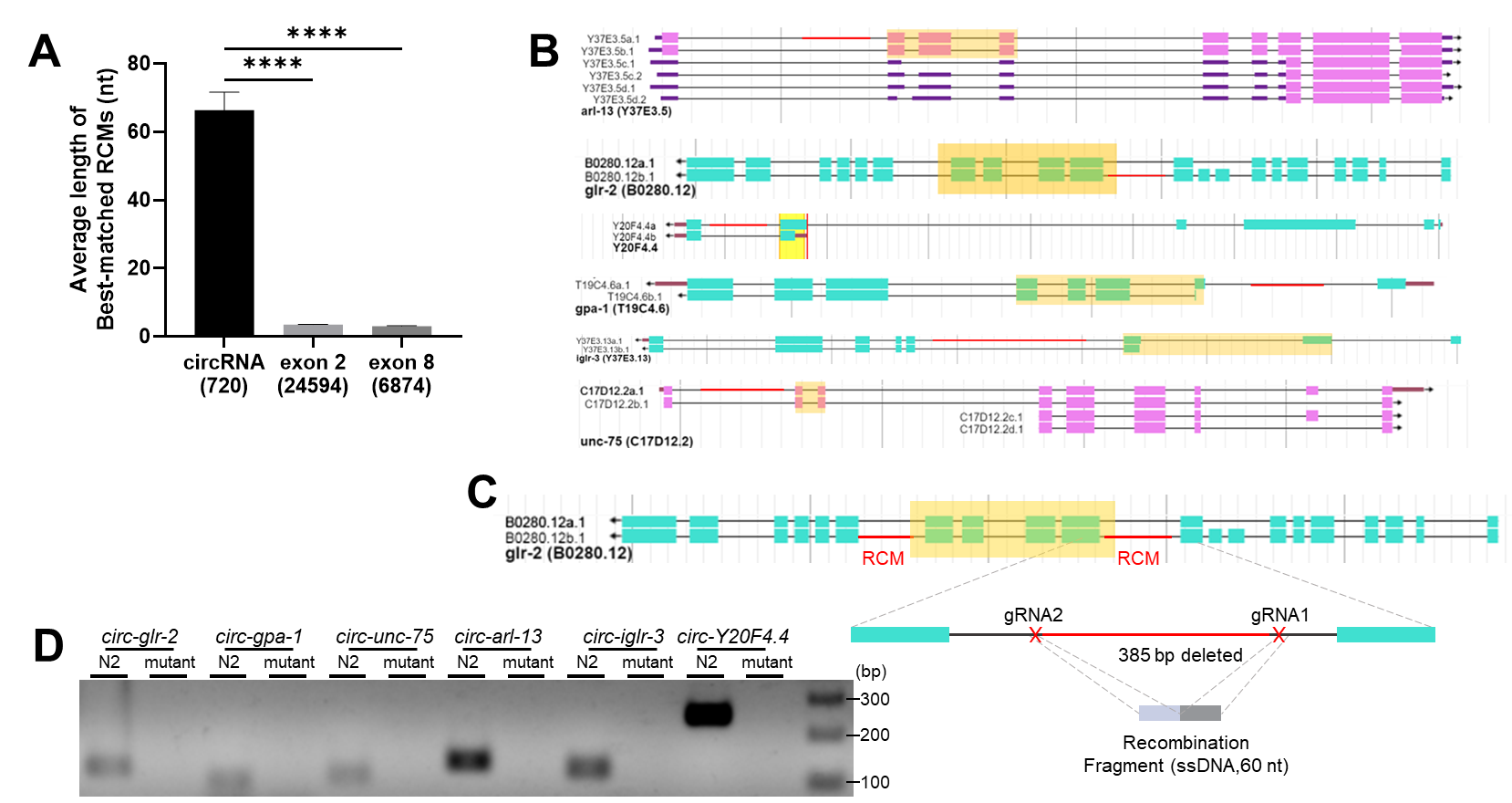


**Figure S4.** circRNA-flanking intron analysis and RCM deletions in circRNA genes.

(A) Lengths of best-matched RCMs in one pair of circRNA-flanking introns compared with those in control exons (exon 2 and exon 8). Values are shown as mean ± SEM. Numbers in the brackets are numbers of intron pairs for analysis. *p* values are from Kruskal-Wallis test with Dunn’s post-hoc test for multiple comparisons. ****, *p* < 0.0001.

(B) Positions of deleted RCMs (red line) of the 6 circRNA genes. Exons in orange shadows are to form circRNAs.

(C) Illustration of RCM deletion in *glr-2*. Red lines in introns are RCMs. Red crosses denote gRNA positions.

(D) Cropped gel image of amplification of circRNA using divergent primers in wild-type N2 strain and RCM-deletion strains (mutant) of 6 circRNA genes.


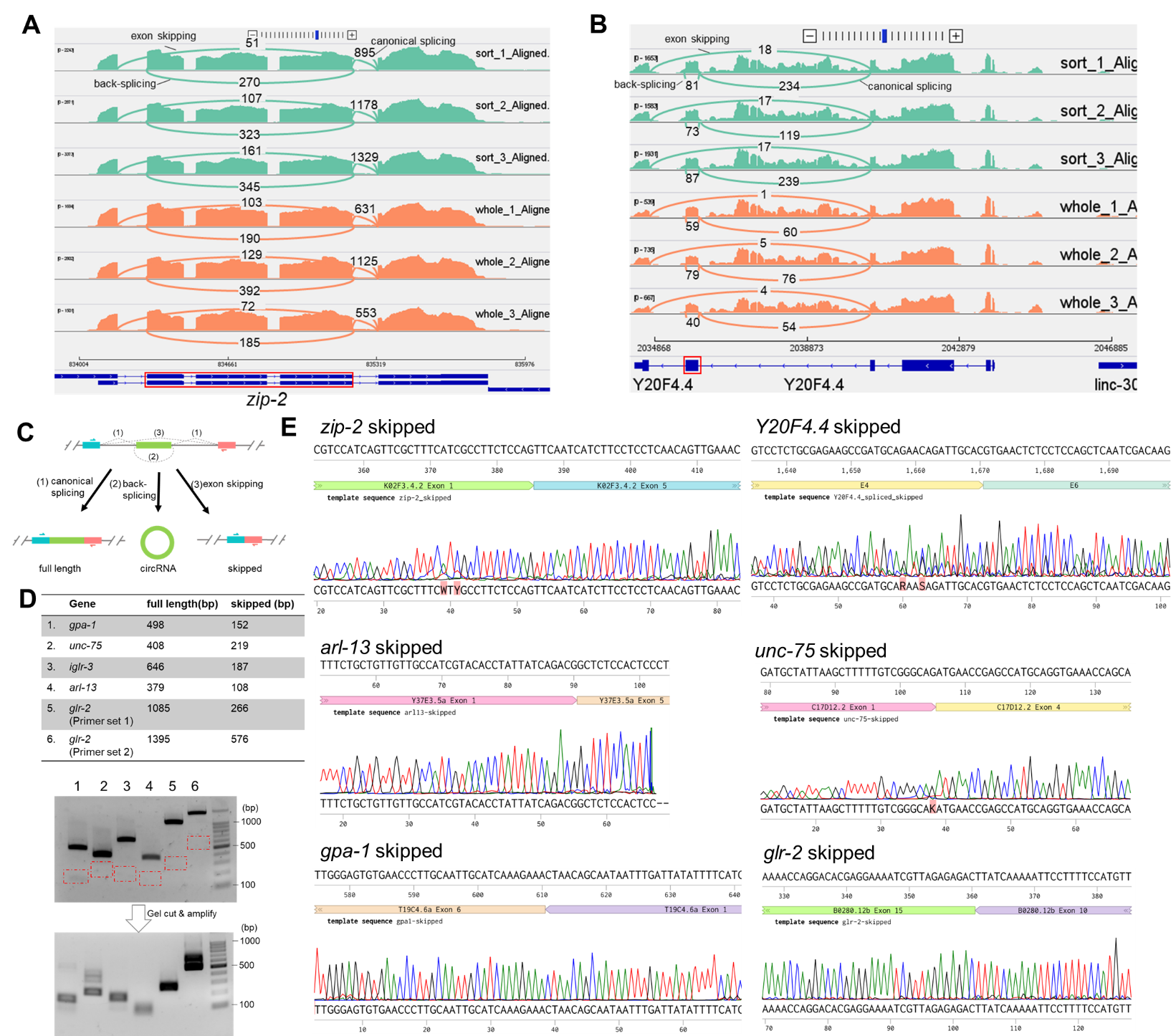


**Figure S5.** Skipped transcripts in several circRNA genes.

(A & B) Sashimi plots showing the number of reads that aligned to the junction of back-splicing, canonical splicing, and exon-skipping in *zip-2* and *Y20F4.4* in the RNA-seq dataset of this study. Exon(s) in red rectangles are circularized to form circRNAs.

(C) Illustration of a circRNA-producing gene producing three transcripts: full-length, circular and skipped. Primers used to detect both the full-length and the skipped transcripts are shown.

(D) Amplification of the skipped transcripts from several circRNA genes by 2-round PCRs. Red rectangles mark the gel areas to be cut. Gel images were cropped for clarity.

(E) Confirmation of sequences of the skipped transcripts in 6 circRNA genes.


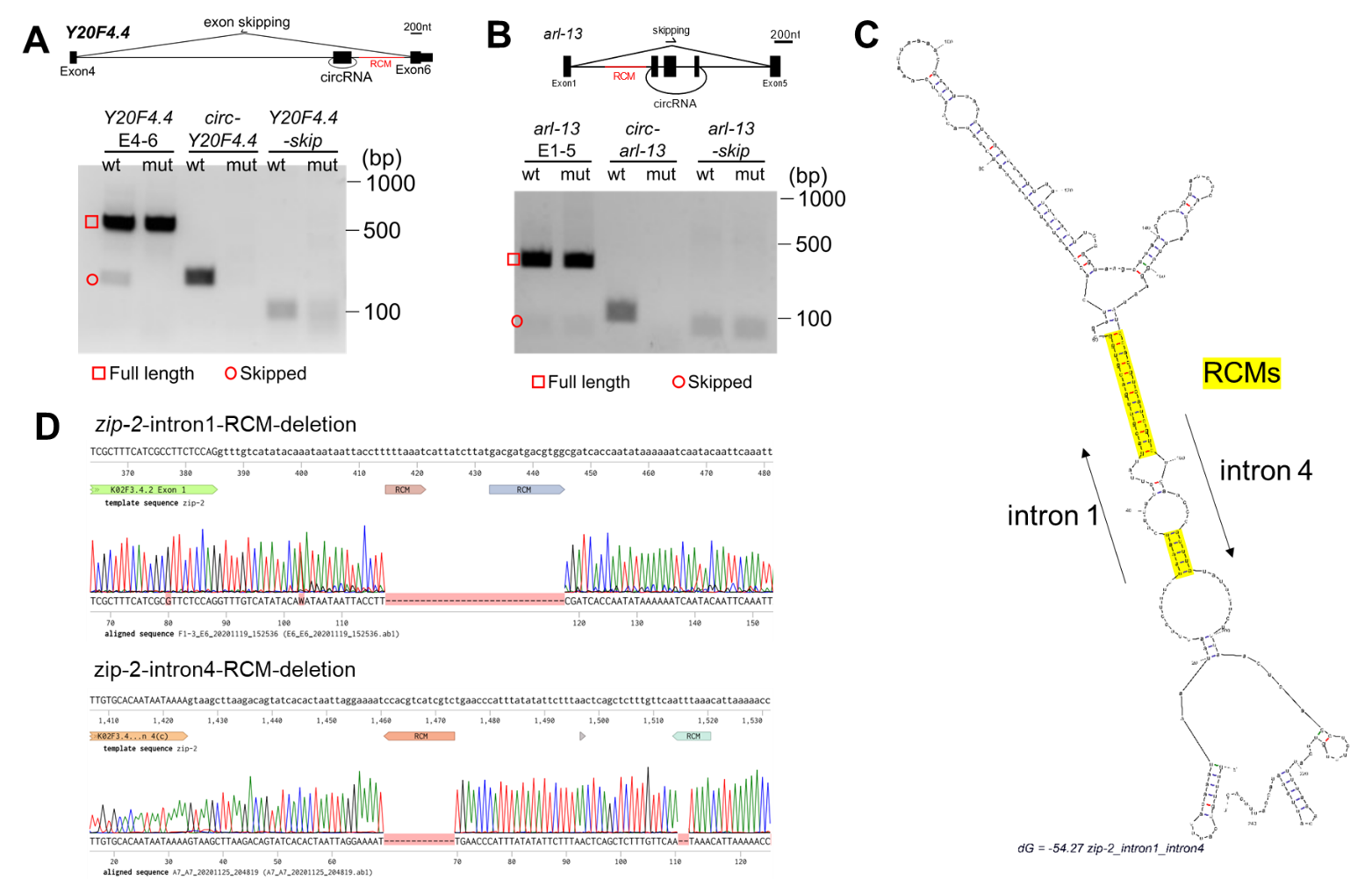


**Figure S6.** Effect of RCM-deletion on exon-skipping.

(A) RT-PCR detection of *Y20F4.4* transcripts in wild-type N2 strain (wt) and the RCM-deleted *Y20F4.4* strain (mut). Gel image was cropped for clarity.

(B) RT-PCR detection of *arl-13* transcripts in wild-type N2 strain (wt) and the RCM-deleted *arl-13* strain (mut). Gel image was cropped for clarity.

(C) Folding prediction of intron 1 and intron 4 of *zip-2* by Mfold (<http://www.unafold.org/mfold/applications/rna-folding-form.php>). RCM sequences are highlighted.

(D) Deleted RCM sequences in intron 1 and intron 4 of *zip-2*.


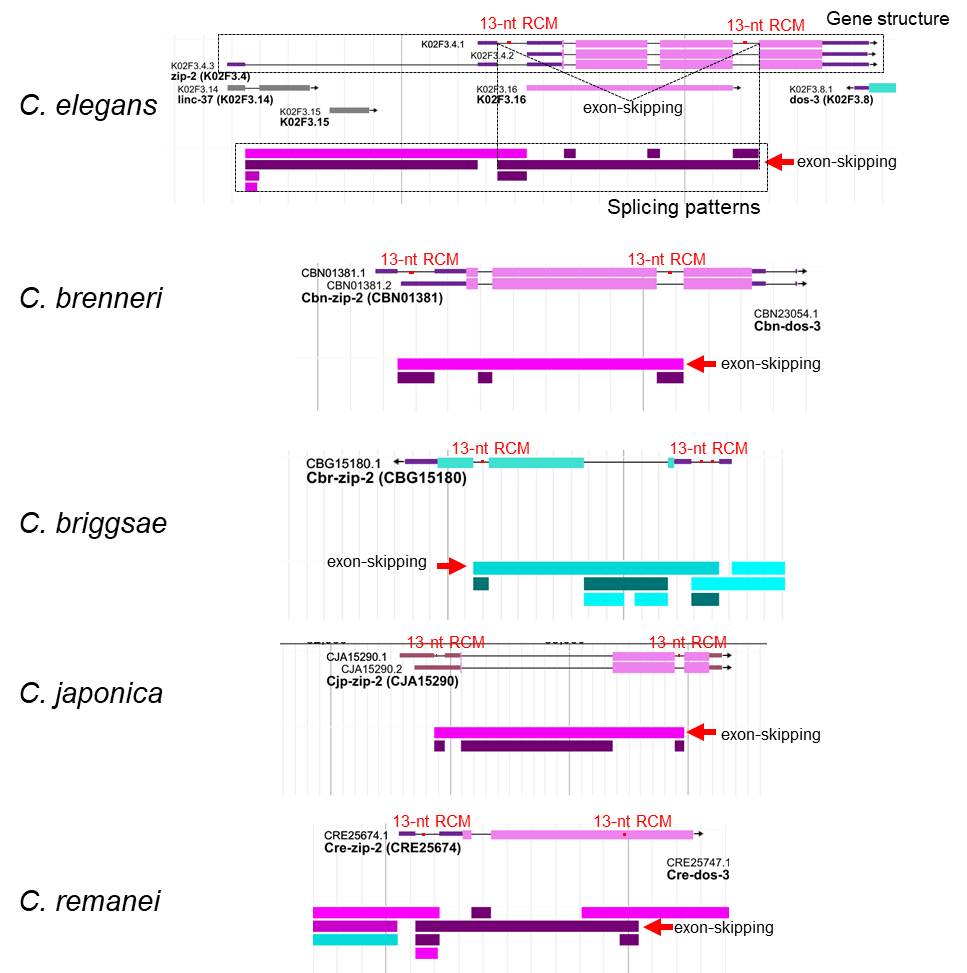


**Figure S7.** Gene structures of *zip-2* ortholog genes.

Gene structures of ortholog *zip-2* genes in indicated nematode species are shown. The splicing patterns of these genes are also shown (from WormBase), with rectangles indicating the joining of 5’ss and 3’ss. The conserved 13-nt RCMs are labeled. Red arrows indicate the splice junctions of the skipped transcripts. Note that the RCMs are always near the joining sites of exon-skipping in these genes. There are two copies of RCM sequences in the upstream intron of *Cbr-zip-2*.


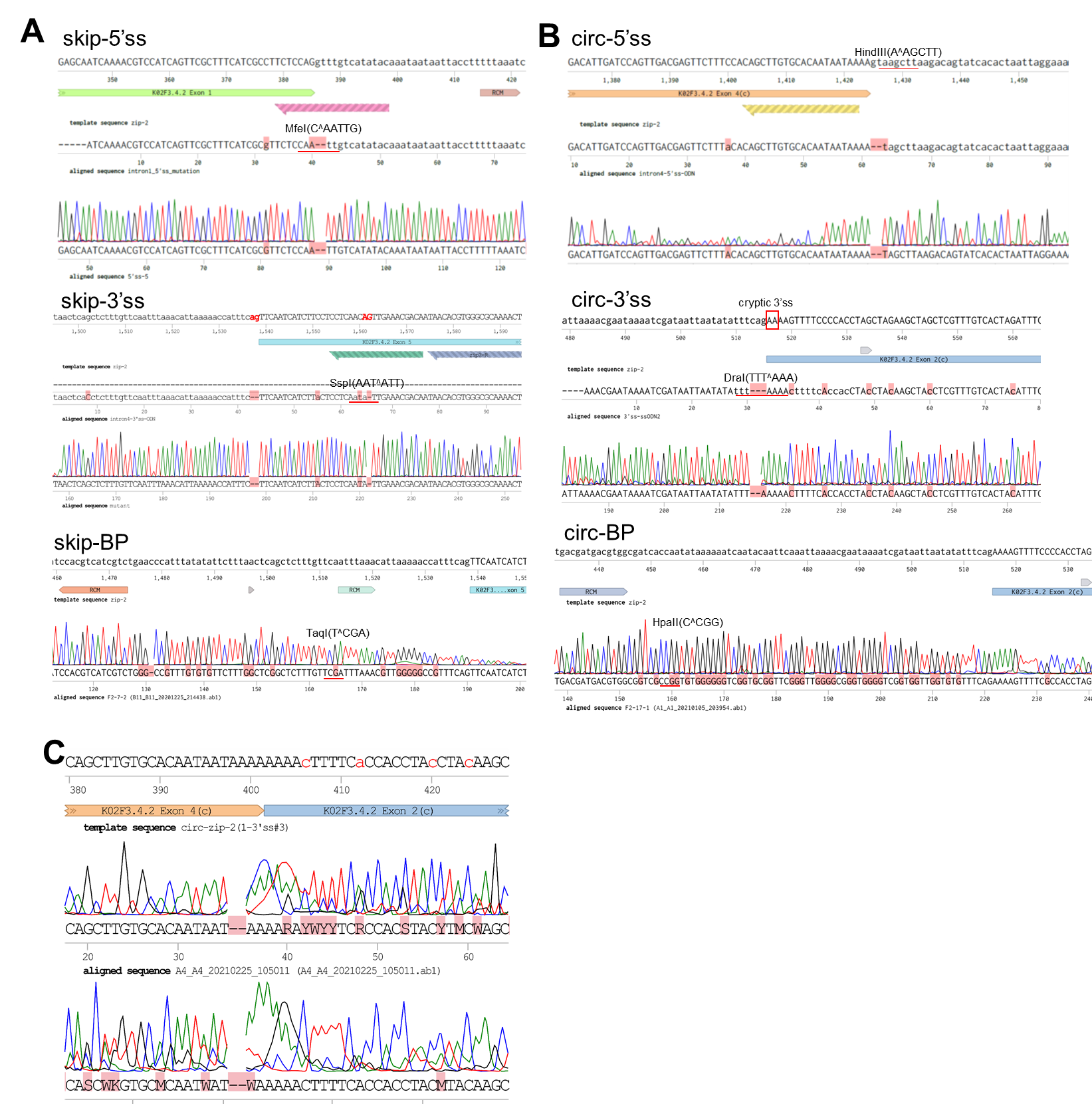


**Figure S8.** Sequence confirmation of mutated ss and BP sites in *zip-2*.

(A, B) Sanger sequence results of splicing sites and branch points mutation in intron 1 and intron 4 of *zip-2*. The enzyme digestion sites used to distinguish wild-type sequences and mutated sequences are labeled. The position of the cryptic 3’ss in circ-3’ss mutation is labeled.

(C) Sanger sequence of *circ-zip-2* produced from the *zip-2*(circ-3’ss) strain. Note that amplified sequences are 2 nt shorter than the predicted BSJ sequences.


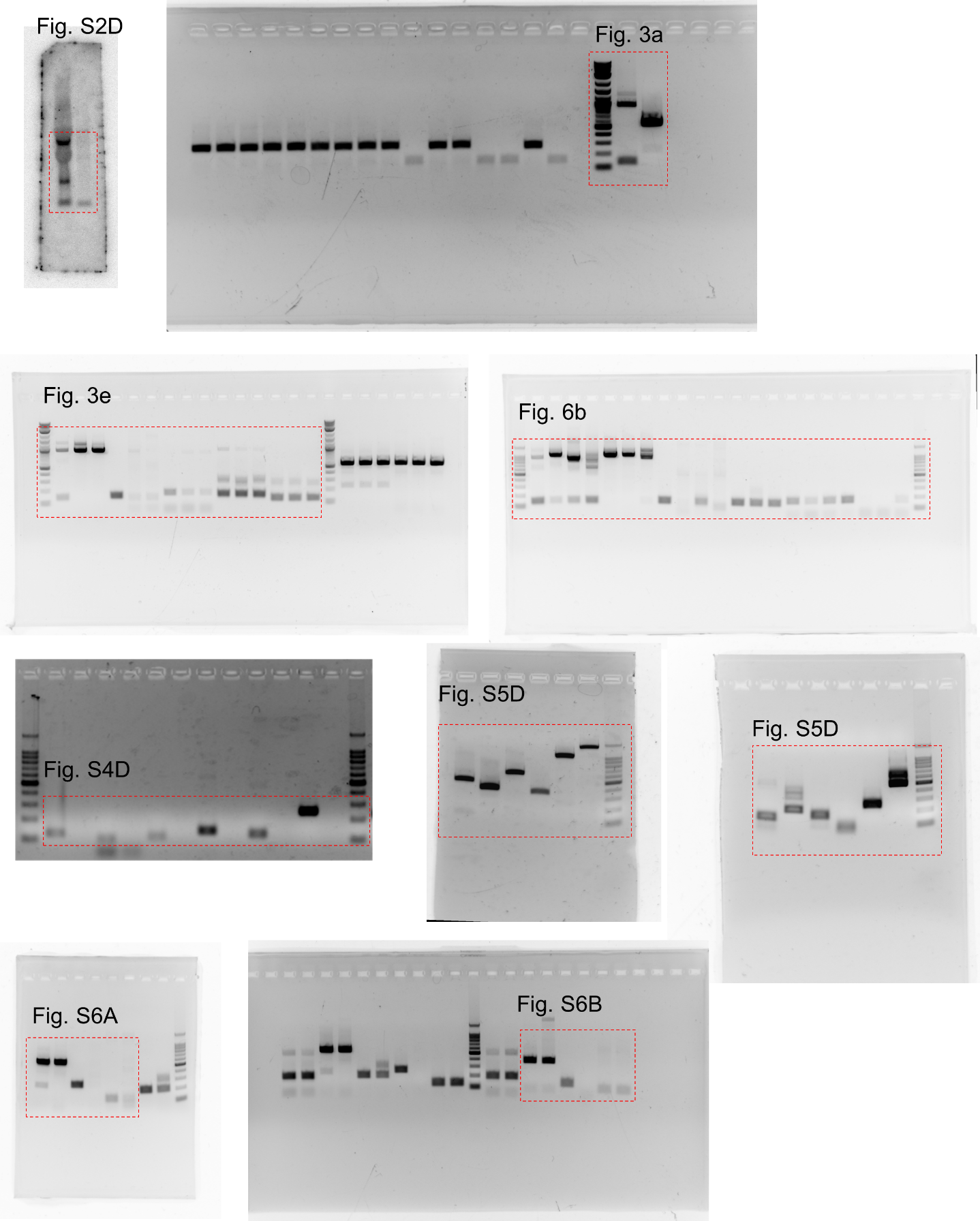


**Figure S9.** Original full images of northern blot and agarose gels. The corresponding figure numbers are labeled. Kept areas are in red rectangles.

**References:**

1. M. Cortes-Lopez *et al.*, Global accumulation of circRNAs during aging in *Caenorhabditis elegans*. *BMC Genomics* **19**, 8 (2018).
